# Muscle contractile properties and perceived fatigue in the general and diseased population

**DOI:** 10.14814/phy2.70134

**Published:** 2024-12-11

**Authors:** Isa H. Mast, Neeltje A. E. Allard, Dominique ten Haaf, Anouk A. F. Stoffels, Lando Janssen, Hieronymus W. H. van Hees, Silvie Timmers, Bregina T. P. Hijmans‐Kersten, Maria T. E. Hopman, Laurien M. Buffart

**Affiliations:** ^1^ Department of Medical BioSciences Radboud University Medical Center Nijmegen The Netherlands; ^2^ FrieslandCampina Amersfoort The Netherlands; ^3^ Department of Pulmonary Diseases Radboud University Medical Center Nijmegen The Netherlands; ^4^ Human and Animal Physiology Wageningen, University & Research Wageningen The Netherlands

**Keywords:** diseased population, fatigability, muscle function, muscle relaxation times, perceived fatigue

## Abstract

Knowledge of muscle contractile properties, physical fitness, and their associations with perceived fatigue may provide insights into mechanisms inducing fatigue and treatment targets. We aimed to identify differences in contractile properties and physical fitness between populations, and examine associations with perceived fatigue. We pooled data on perceived fatigue, physical fitness, and contractile properties from six studies, including a control group (*n* = 90), cancer survivors (*n* = 27), patients with chronic obstructive pulmonary disease (COPD; *n* = 16), chronic myeloid leukemia (CML; *n* = 20), and statin users (*n* = 64). We evaluated differences between populations, and associations of contractile properties and physical fitness with perceived fatigue. Compared with the control group, we found differences in contractile properties of patients with COPD (larger muscle force decline: *β* = −10.5%, 95% CI = −16.7; −4.2, increase in early relaxation time (Rt): *β* = 84.4%, 95% CI = 51.7; 117.0, increase in half Rt: *β* = 83.1%, 95% CI = 45.5; 120.7, muscle force rise (MFR): *β* = 0.2%/ms, 95% CI = 0.1; 0.3, and decrease in MFR: *β* = −24.3%, 95% CI = −35.7; −13.0) and statin users (early Rt: *β* = −5.4 ms, 95% CI = −10.0; −0.8, increase in early Rt: *β* = 19.8%, 95% CI = 2.5; 37.1). Associations between contractile properties and perceived fatigue varied across populations. Longer relaxation times were associated with higher perceived fatigue in hemato‐oncological populations. To conclude, contractile properties were impaired in patients with COPD and statin users. Associations between contractile properties and perceived fatigue varied across populations. In hemato‐oncological populations, impaired muscle relaxation was associated with higher perceived fatigue.

## 1 INTRODUCTION

Fatigue is a commonly reported (patho)physiological symptom in the general and diseased population (Yoon et al., 2023). Fatigue can be defined as a subjective sense of tiredness, weakness, or exhaustion, either physically, mentally, or both (Finsterer & Mahjoub, 2014; Landmark‐Høyvik et al., 2010), and is frequently reported by patients in primary and community care with a prevalence up to 8% (Nicholson et al., 2015). Furthermore, fatigue can occur during chronic diseases including cancer, cardiovascular disease, or chronic obstructive pulmonary disease (COPD) and their associated treatments (for example statins, radiotherapy, or systemic cancer treatments (Finsterer & Mahjoub, 2014; Thompson et al., 2016)). It is often reported as one of the most severe and distressing symptoms impairing daily functioning and health‐related quality of life (HRQoL) (Dittner et al., 2004; Janssen et al., 2003).

Assessments of fatigue are often divided into perceived fatigue evaluated by self‐reported questionnaires, and performance fatigability (Kluger et al., 2013). Performance fatigability can be quantified by the decline in muscle force‐producing capacity during a prolonged task (Finsterer & Mahjoub, 2014; Kluger et al., 2013), and can be related to impaired central activation of α‐motorneurons or impaired muscle contractile properties (Allen et al., 2008). Electrical stimulation can be used to assess muscle contractile properties independent of a patient's motivation or effort, bypassing central activation (Zwarts et al., 2008). The relationship between perceived fatigue and performance fatigability is complex and studies examining this relationship have primarily focussed on homogeneous study populations (Kluger et al., 2013), hampering direct comparisons between populations.

Treatment strategies for perceived fatigue include among others, pharmacological interventions, exercise interventions, and/or psychological interventions (Finsterer & Mahjoub, 2014). However, the effectiveness of these interventions on perceived fatigue is generally small and varies within and between populations (Barakou et al., 2023; Finsterer & Mahjoub, 2014). Understanding mechanisms underlying fatigue such as physical fitness or muscle contractile properties may help identify targets for therapeutic interventions. Altered muscle contractile properties such as slowed muscle relaxation or maximal force rise might reflect underlying changes in muscle fiber‐type composition, energy metabolism, or calcium handling, which can significantly impact exercise performance and contribute to fatigue (Grassi et al., 2015). Knowledge on associations between physiological muscle properties and perceived fatigue across diverse populations might help to advance the understanding of potential differences in the etiology of fatigue and the development of targeted treatment strategies to reduce fatigue.

Hence, in this study, we aimed to identify differences in muscle contractile properties and physical fitness between different patient populations and a control group. Furthermore, we aimed to examine associations of muscle contractile properties and physical fitness with perceived fatigue for the total population and to examine the differences between subgroups.

## 2 MATERIALS AND METHODS

We pooled data on perceived fatigue, physical fitness, and muscle contractile properties from six studies conducted in the Radboud University Medical Center (Allard et al., 2021, 2023; Janssen et al., 2019; Mast et al., 2024; Stoffels et al., 2024; Ten Haaf et al., 2019) using the same electrical stimulation protocol, containing a control group (*n* = 90) and different patient populations (*n* = 127), including 27 cancer survivors with cancer‐related fatigue, 16 patients with moderate‐to‐severe chronic pulmonary obstructive disease (COPD), 20 patients with chronic myeloid leukemia (CML) treated with tyrosine kinase inhibitors (TKI), and 64 patients who use statins for risk reduction of cardiovascular disease (Table 1). Data about muscle contractile properties, questionnaires about perceived fatigue, anthropometrics and aerobic fitness was collected from all six studies, if available (Allard et al., 2021, 2023; Janssen et al., 2019; Mast et al., 2024; Stoffels et al., 2024; Ten Haaf et al., 2019) (Table 1). Details on disease severity and in‐ and exclusion criteria can be found in the original articles (Allard et al., 2021, 2023; Janssen et al., 2019; Mast et al., 2024; Stoffels et al., 2024; Ten Haaf et al., 2019). All patients provided written informed consent, and all studies have been approved by the local medical ethics committee (METC Oost‐Nederland, formerly known as CMO region Arnhem‐Nijmegen) (Allard et al., 2021, 2023; Janssen et al., 2019; Mast et al., 2024; Stoffels et al., 2024; Ten Haaf et al., 2019).

**TABLE 1:** Descriptive information of studies using electrical muscle stimulation and the included population subgroups.

First author (year)	Study population	*N*	Sex female, *n* (%)	Age, mean ± SD (years)	Fatigue question naire	Aerobic fitness assessment (PeakVO_2_)
Ten Haaf et al. (2019)	Controls	52	5 (20)	70 ± 4	–	Ästrand‐Rhyming test
Allard et al. (2021)	Statin users	32	9 (28)	64 ± 4	BFI	CPET
	Controls	20	10 (50)	63 ± 5		
Allard et al. (2023)	Statin users	32	10 (31)	63 ± 7	BFI	–
	Controls	18	7 (39)	66 ± 6		
Janssen et al. (2019)	Patients with CML	20	6 (30)	54 ± 8	BFI	CPET
Mast et al. (2024)	Cancer survivors	27	10 (37)	59 ± 15	CIS	Ästrand‐Rhyming test
Stoffels et al. (2024)	Patients with COPD	16	10 (63)	61 ± 8	CIS	CPET

Abbreviations: BFI, brief fatigue inventory; CIS, checklist individual strength; CML, chronic myeloid leukemia; COPD, chronic obstructive pulmonary disease; CPET, cardiopulmonary exercise test; SD, standard deviation.

### 2.1 Perceived fatigue

Perceived fatigue was measured using the checklist individual strength (CIS) (Mast et al., 2024; Stoffels et al., 2024) or the brief fatigue inventory (BFI), if available (Allard et al., 2021, 2023; Janssen et al., 2019) (Table 1). The subscale fatigue of the CIS questionnaire consists of eight items scored on a 7‐point Likert scale, with scores ranging between 8 and 56 (Vercoulen et al., 1994). The BFI consists of nine items scored on an 11‐point Likert scale, and yields a total score by averaging all items (Mendoza et al., 1999). Higher scores on the CIS and BFI questionnaires correspond to higher levels of perceived fatigue. To allow for pooling of these questionnaires, individual scores were normalized to a 0 to 100 scale by linear transformation.

### 2.2 Muscle contractile properties

Muscle contractile properties of the dominant *Quadriceps femoris* muscle were determined using the same electrical stimulation protocol and same stimulation parameters in all six studies (Allard et al., 2018; Mast et al., 2024). The muscle electrical stimulation protocol is extensively described elsewhere (Allard et al., 2018; Mast et al., 2024). In short, participants were seated in upright position, and surface electrodes were placed on the distal and proximal part of the anterior thigh. The protocol included evaluations of maximal voluntary contraction (MVC) assessed by maximally extending the knee during an isometric contraction for at least 3 s and calculating the mean maximal force (N) over a stable interval of approximately 1 s. Subsequently, the muscle was electrically stimulated by inducing a force of at least 40% of the MVC for 1 s with a frequency of 50 Hz. Muscle fatigability was evaluated by a 2‐min protocol repetitively using bursts of 30 Hz of 1 s duration every 2 s (Allard et al., 2018; Mast et al., 2024).

Force signals were analyzed using Matlab (Version R2022a; The MathWorks Inc., Natick, Massachusetts). Muscle force decline was used as an indicator for muscle fatiguability, and was evaluated as the percentage peak force decline between the first and last three bursts of the fatigability protocol, $\text{Formula}\;1=\left(\frac{\text{mean last three bursts}-\text{mean first three bursts}}{\text{mean first three bursts}}\right)*100$. Early‐ and half relaxation time (Rt) during a single burst were defined as the time needed for the force to decline from 75% to 50% and from 50% to 25% of peak force, respectively, averaged over the first three bursts of the fatigability protocol. Maximal force rise (MFR) was calculated as the percentage of maximal force incline per millisecond divided by the peak force, averaged over the first three bursts of the fatigability protocol. The increase or decrease in relaxation times or MFR was calculated based on the change between the first and last three bursts of the fatigability protocol (Formula 1; (Allard et al., 2018)). The coefficient of variation (CV) of mean signals during the fatiguability protocol was calculated as CV = $\frac{\sigma }{\mu }*100$, with σ representing the average standard deviation and μ the average mean across all timepoints during the fatiguability protocol.

### 2.3 Aerobic fitness

Aerobic fitness was measured using the Ästrand‐rhyming test (Mast et al., 2024; Ten Haaf et al., 2019) or a cardiopulmonary exercise test (CPET) on a cycle ergometer (Allard et al., 2021; Janssen et al., 2019; Stoffels et al., 2024) (Table 1). The Ästrand‐rhyming test is a submaximal exercise test including 6‐min steady state exercise at a target heart rate of *180—age*. PeakVO_2_ was estimated based on the steady‐state workload and mean heart rate of the 5th and 6th minute of the test (Mast et al., 2024). The CPET protocol included ramp‐incremental maximal exercise with an increasing workload of 10–15 Watt/min, and peakVO_2_ was determined as an average of the highest 30 s of VO_2_ uptake (Allard et al., 2018).

### 2.4 Demographic and anthropometric information

Information about age and sex was collected from all studies. In addition, measured body height and weight were collected from all studies and body mass index (BMI) was calculated in kg/m^2^.

### 2.5 Statistical analysis

Statistical analyses were performed using RStudio (R Core Team). Descriptives were generated for anthropometric and demographic variables and parameters of perceived fatigue and performance fatigability. Multiple linear regression models were used to examine differences in contractile properties between populations and to examine the association between muscle contractile properties and perceived fatigue, adjusting for age, sex, and study. To explore potential differences in this association between populations, we added an interaction term between muscle property and population subgroup into the model. In case the interaction term significantly improved the model, as determined by the likelihood ratio test, stratified analyses were conducted to explore associations separately within each population subgroup, adjusted for age and sex. Considering the power of our analyses, we did not include multiple measures fitness or muscle contractile properties simultaneously in our models. Assumptions of linearity, normality of residuals, and homoscedasticity were checked for all models. Regression coefficients (β), and 95% confidence intervals (CI) were presented. Statistical significance was set at *p* < 0.05.

## 3 RESULTS

### 3.1 Patient characteristics

Two hundred and seventeen participants were included in the analyses. Mean age of the total sample was 63.7 ± 8.9 years, participants had a mean BMI of 26.4 ± 3.9 kg/m^2^, and 38% was female (Table 2).

**TABLE 2:** Descriptive information on demographics, perceived fatigue, physical fitness and muscle contractile properties of the total population and population subgroups.

	Total population (*n* = 217)	Control group^a^ (*n* = 90)	Cancer Survivors (*n* = 27)	Patients with CML (*n* = 20)	Patients with COPD (*n* = 16)	Statin users (*n* = 64)
**Sex**, female, *n* (%)	83 (38)	26 (29)	10 (37)	6 (30)	10 (62)	31 (48)
**Age**, mean ± SD	63.7 ± 8.9	67.6 ± 5.8	59.3 ± 15.0	54.2 ± 8.4	61.2 ± 8.0	63.6 ± 5.7
**Body height** (cm), mean ± SD	175.1 ± 8.8	176.4 ± 8.1	173.7 ± 6.3	176.5 ± 10.9	170.0 ± 11.0	174.9 ± 9.1
**Body weight** (kg), mean ± SD	81.3 ± 14.3	82.0 ± 12.6	80.8 ± 14.3	80.2 ± 13.6	73.8 ± 17.4	82.9 ± 15.7
**Body Mass Index** (kg/m^2^), median [IQR]	26.4 ± 3.9	26.3 ± 3.2	26.9 ± 5.0	25.8 ± 4.1	25.1 ± 5.3	27.0 ± 3.8
**CIS score**, mean ± SD	34.3 ± 9.6		34.7 ± 9.0		33.6 ± 10.8	
**BFI score**, mean ± SD	1.5 ± 1.8	1.0 ± 1.4		2.3 ± 2.2		1.5 ± 1.7
**Perceived fatigue** (0–100), mean ± SD	25.2 ± 25.3	9.9 ± 14.0	**55.6 ± 18.7** *	23.3 ± 21.8	**53.3 ± 22.6** *	15.0 ± 17.4
**PeakVO**_**2**_ (mL/kg/min), mean ± SD	32.3 ± 11.4	37.6 ± 11.5	**27.5 ± 8.1** *	34.6 ± 8.4	**15.6 ± 3.8** *	**31.3 ± 7.3** *
**Maximal MVC** (N), mean ± SD	597.0 ± 177.9	637.3 ± 177.2	**484.9 ± 131.6** *	660.5 ± 189.2	**429.3 ± 138.6** *	610.7 ± 158.7
**MVC per kg bodyweight** (N/kg), mean ± SD	7.4 ± 1.9	7.9 ± 1.8	**6.1 ± 1.8** *	8.3 ± 2.0	**5.9 ± 2.0** *	7.4 ± 1.7
**Muscle force decline** (%), mean ± SD	−30.7 ± 10.2	−29.4 ± 10.0	−29.3 ± 12.2	−31.8 ± 8.7	**−35.9 ± 11.3** *	−31.3 ± 9.4
**Half relaxation time** (ms), median [IQR]	33.5 [30.2–38.1]	33.0 [30.7–36.7]	36.0 [28.7–39.3]	32.3 [29.0–41.2]	32.0 [29.5–36.2]	33.7 [31.6–37.2]
**Increase in half Rt** (%), median [IQR]	91.7 [62.2–127.5]	87.3 [60.8–132.5]	70.2 [41.9–109.6]	86.2 [48.0–101.9]	**151.5 [118.0**–**218.2]** *	92.0 [69.3–111.4]
**Early Rt** (ms), median [IQR]	24.0 [22.0–26.2]	24.7 [22.7–26.7]	23.0 [21.2–26.2]	22.5 [20.6–24.5]	22.7 [20.5–24.3]	**24.3 [22.2**–**26.3]** *
**Increase early Rt** (%), median [IQR]	82.4 [57.6–115.2]	76.2 [45.1–102.4]	77.6 [58.9–114.3]	86.3 [53.8–100.4]	**157.3 [98.6**–**199.8]** *	**82.8 [57.6**–**118.7]** *
**MFR** (%/ms), mean ± SD	0.8 ± 0.2	0.8 ± 0.2	0.8 ± 0.2	0.7 ± 0.1	**0.9 ± 0.2** *	0.8 ± 0.1
**Decrease in MFR** (%), median [IQR]	−14.2 [−28.8 to −7.1]	−12.5 [−26.7 to −7.4]	−7.2 [−16.5 to 3.2]	−14.1 [−22.8 to −6.0]	**−33.9 [−41.7** to **−27.8]** *	−18.0 [−29.9 to −9.7]

Abbreviations: BFI, brief fatigue inventory; CIS, checklist individual strength; CML, chronic myeloid leukemia; COPD, chronic obstructive pulmonary disease; MFR, Maximal Force Rise; MVC, maximal voluntary contraction; PeakVO_2_, maximal oxygen uptake; Rt, relaxation time.

^a^ Fatigue was available for 38 participants in the control group.

* Statistically significant difference from control group. Differences are tested using linear regression models corrected for age, sex and study allocation.

### 3.2 Perceived fatigue

Normalized perceived fatigue score of the total population was 25.2 ± 25.3 (Table 2). Perceived fatigue was significantly higher in cancer survivors (*β* = 37.9, 95% CI = 28.8; 46.9) and patients with COPD (*β* = 34.7, 95% CI = 24.2; 45.2), compared to the control group (Table S1).

### 3.3 Physical fitness and muscle contractile properties

**FIGURE 1:**
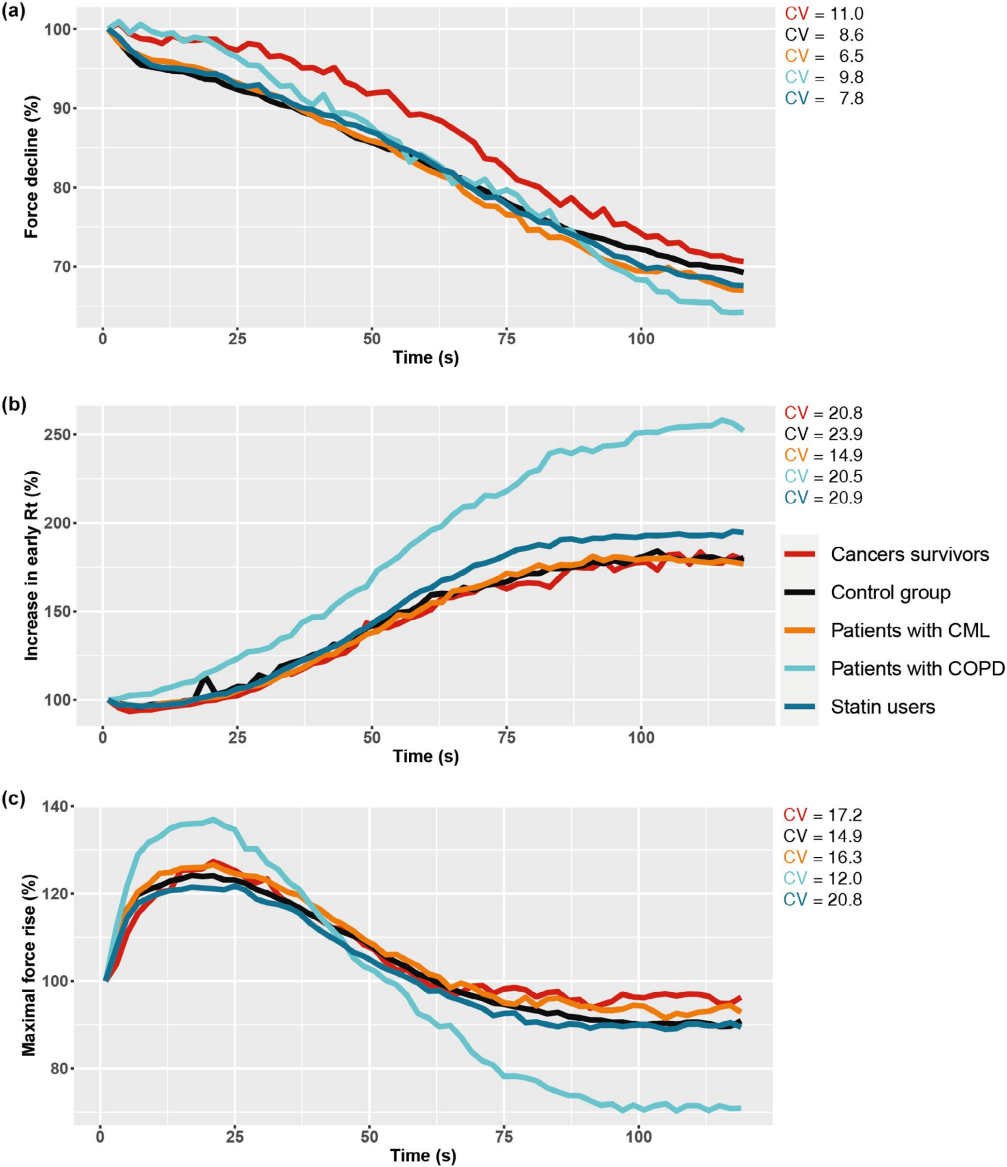
Average signals representing the fatigability protocol in each of the population subgroups, with their coefficient of variation (CV) presented in the legend. Panel (a) shows muscle force decline, which presents a larger decline during the fatigability protocol in patients with COPD (light blue) compared to the control group (black). Panel (b) shows a larger increase in early relaxation time during the fatigability protocol in statin users (dark blue) and patients with COPD (light blue) compared to the control group (black), Panel (c) shows maximal force rise, which shows a larger reduction during the fatigability protocol in patients with COPD (light blue) compared to the control group (black).

(Estimated) PeakVO_2_ was 32.3 ± 11.4 mL/kg/min for the total group (Table 2). PeakVO_2_ was lower in cancer survivors (*β* = −9.9 mL/kg/min, 95% CI = −15.7; −4.1), patients with COPD, (*β* = −20.9 mL/kg/min, 95% CI = −27.2; −14.6), and statin users (*β* = −5.5 mL/kg/min, 95% CI = −11.0; −0.1) compared to the control group (Table 2; Figure 1; Table S1). Additionally, MVC was lower in cancer survivors (MVC, *β* = −129.8 N, 95% CI = −211.9; −47.8, MVC per kg bodyweight, *β* = −1.8 N, 95% CI = −2.7; −0.9) and in patients with COPD (MVC, *β* = −148.3 N, 95% CI = −243.3; −53.4, MVC per kg bodyweight, *β* = −1.7 N, 95% CI = −2.8; −0.7) compared to the control group (Table 2; Table S1).

Muscle contractile properties of the total population and of the subpopulations are presented in Table 2. Patients with COPD showed a larger muscle force decline (*β* = −10.5%, 95% CI = −16.7; −4.2; Figure 1a), larger increase in early Rt (*β* = 84.4%, 95% CI = 51.7; 117.0; Figure 1b), larger increase in half Rt (*β* = 83.1%, 95% CI = 45.5; 120.7), and a larger decrease in MFR (*β* = −24.3%, 95% CI = −35.7; −13.0; Figure 1c) between the first and last bursts of the fatiguability protocol, compared to the control group (Table S1). Additionally, patients with COPD had a faster MFR at onset of the fatiguability protocol (*β* = 0.2%/ms, 95% CI = 0.1; 0.3; Figure 1c), compared to the control group (Table S1). A smaller early Rt at onset of the fatiguability protocol (*β* = −5.4 ms, 95% CI = −10.0; −0.8) and larger increase in early Rt between the first and last bursts of the fatiguability protocol were found in statin users compared to the control group (*β* = 26.2%, 95% CI = 4.3; 48.2; Figure 1b; Table S1).

### 3.4 Associations of physical fitness and muscle contractile properties with perceived fatigue

**FIGURE 2:**
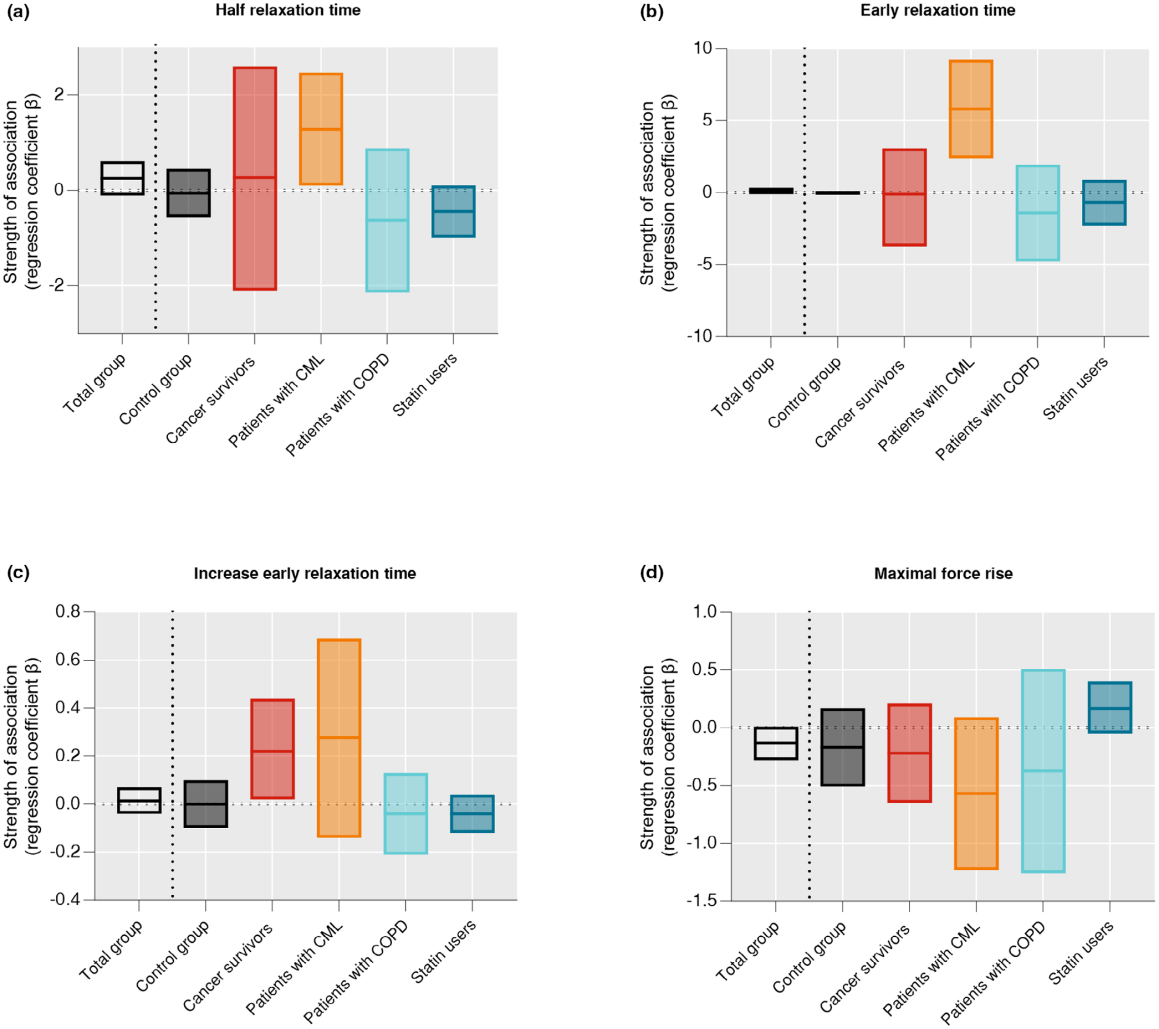
Visual representation of the association represented by the regression coefficient (*β*) and upper and lower bounds of the 95% confidence interval (CI), between (a) half relaxation time, (b) early relaxation time, (c) increase in early relaxation time, and (d) maximal force rise with fatigue across the total population and within population subgroups.

For the total population, physical fitness and muscle contractile properties were not significantly associated with perceived fatigue (Table 3). However, the associations of early‐ and half relaxation times, increase in early relaxation time, and decrease in MFR with perceived fatigue differed between populations (Table 3; Figure 2). In patients with CML, longer early (*β* per ms = 1.28, 95% CI = 0.10; 2.47) and half relaxation times (*β* per ms = 5.81, 95% CI = 2.39; 9.23) were significantly associated with higher perceived fatigue. In cancer survivors, a larger increase early Rt between the first and last bursts of the fatiguability protocol was associated with higher perceived fatigue (*β* per % = 0.23, 95% CI = 0.02; 0.44). These associations were not statistically significant in the other populations. The association between MFR and perceived fatigue differed between populations, however, none of the associations within population subgroups reached statistical significance (Table 3).

**TABLE 3:** Associations of physical fitness and contractile properties with perceived fatigue for the total population and population subgroups.

			Association with perceived fatigue for the population subgroups				
	Overall association with perceived fatigue	Differences between groups*	Control group (*n* = 38)	Cancer Survivors (*n* = 27)	Patients with CML (*n* = 20)	Patients with COPD (*n* = 16)	Statin users (*n* = 64)
	*β* (95% CI)	*p*‐Value	*β* (95% CI)	*β* (95% CI)	*β* (95% CI)	*β* (95% CI)	*β* (95% CI)
**PeakVO**_**2**_ (mL/kg/min)	−0.22 (−0.76;0.32)	0.550					
**MVC** (N)	−0.01 (−0.03;0.01)	0.158					
**MVC per kg bodyweight** (N/kg)	−0.90 (−2.51;0.71)	0.554					
**Muscle force decline** (%)	0.18 (−0.10;0.46)	0.200					
**Half relaxation time** (ms)	0.25 (−0.11;0.61)	**0.037**	−0.06 (−0.56;0.45)	0.26 (−2.13;2.65)	**1.28 (0.10;2.47)**	−0.63 (−2.14;0.88)	−0.44 (−0.99;0.10)
**Increase in half Rt** (%)	0.04 (−0.02;0.09)	0.469					
**Early Rt** (ms)	0.12 (−0.11;0.34)	**0.010**	−0.02 (−0.15;0.10)	−0.34 (−3.75;3.08)	**5.81 (2.39;9.23)**	−1.42 (−4.77;1.94)	−0.70 (−2.28;0.89)
**Increase early Rt** (%)	0.01 (−0.04;0.07)	**0.011**	0.00 (−0.07;0.06)	**0.23 (0.02;0.44)**	0.28 (−0.14;0.69)	−0.04 (−0.21;0.13)	−0.04 (−0.12;0.04)
**MFR** (%/ms)	3.69 (−15.21;22.59)	0.643					
**Decrease in MFR** (%)	−0.13 (−0.28;0.01)	**0.014**	−0.17 (−0.51;0.17)	−0.22 (−0.65;0.21)	−0.57 (−1.23;0.09)	−0.37 (−1.26;0.51)	0.15 (−0.05;0.40)

*Note*: Associations are tested using linear regression analyses corrected for age and sex.

Abbreviations: BFI, brief fatigue inventory; CIS, checklist individual strength; CML, chronic myeloid leukemia; COPD, chronic obstructive pulmonary disease; MFR, Maximal Force Rise; MVC, maximal voluntary contraction; Rt, relaxation time.

* *p*‐Value of the likelihood ratio test.

## 4 DISCUSSION

Our study evaluated differences in muscle contractile properties, physical fitness and fatigue between specific patient populations and a control group, as well as the association of physical fitness and muscle contractile properties with perceived fatigue. There were three key findings. First, compared with a control group cancer survivors and patients with COPD had a lower muscle strength and aerobic fitness. Second, we found substantial muscle fatigue in patients with COPD and impaired muscle relaxation in statin users compared with a control group. Third, the associations between muscle contractile properties and perceived fatigue were not significant in the total population, and were not uniform across population subgroups. Specifically, impaired muscle relaxation was associated with higher perceived fatigue in hemato‐oncological populations, while this was not the case for other subpopulations.

Our findings showed a reduced aerobic fitness and muscle strength in both patients with COPD and cancer survivors. In patients with COPD, a reduced exercise capacity is a frequently reported systemic manifestation of the disease, impacting physical function, and quality of life (GOLD, 2023). Similarly, in cancer survivors, physical fitness and physical function is often reduced due to the disease or its treatment (Campbell et al., 2012). Patients with CML did not show a reduced physical fitness level in this study, which might be related to treatment of or inclusion criteria of the study. In statin users, we found a reduction in aerobic fitness levels but not in muscle strength, which might be related to a reduced level of physical activity or statin associated muscle symptoms, and highlights the importance of aerobic exercise training for this patient population (Noyes & Thompson, 2017; Thompson et al., 2016). Exercise interventions both incorporating aerobic fitness and muscle strength exercises are recommended in exercise guidelines for patients with COPD and cancer survivors (Bolton et al., 2013; Campbell et al., 2019) and may help to improve aerobic fitness and muscle strength and thereby physical function and quality of life in these patient populations.

The larger muscle fatigue in patients with COPD as compared with the control group was indicated by the larger muscle force decline and slowing in muscle force development during the fatiguability protocol. These changes, as well as the rapid MFR at onset of the fatiguability protocol, might be explained by the increased proportion of fast‐twitch type II relative to slow‐twitch type I muscle fibers, leading to a shift towards a glycolytic muscle metabolism in skeletal muscles of patients with COPD (Gosker et al., 2007). Additionally, the observed slowing in relaxation times during the fatiguability protocol in both patients with COPD and statin users might be due to impaired mitochondrial activity or sarcoplasmic reticulum Ca^2+^ handling in skeletal muscles, and might subsequently lead to poor exercise performance we and others found in these populations (Allard et al., 2018; Liantonio et al., 2007; Qaisar et al., 2022). In statin users, altered early relaxation times compared to the control group might be related to statin‐induced myalgia (Allen et al., 2008; Liantonio et al., 2007; Thompson et al., 2016). Notably, despite pronounced alterations, muscle contractile properties in these patient populations were not associated with perceived fatigue, suggesting involvement of other mechanisms in the perception of fatigue such as a limited ventilatory capacity, disrupted mechanisms of central activation, or psychosocial factors in patients with COPD (Ebadi et al., 2021) or muscle pain and cramps in statin users (Thompson et al., 2016).

Our finding that the associations between muscle contractile properties and perceived fatigue was not uniform, suggesting differential pathophysiological mechanisms underlying fatigue perception. We found that impaired contractile muscle properties, particularly muscle relaxation, may potentially contribute to perceived fatigue in hemato‐oncological populations, while this is less likely in the control group, patients with COPD, and statin users. Slowed relaxation times might be a manifestation of muscle fatigue in these patient populations (Westerblad et al., 2010). This suggests that improving muscle relaxation times may potentially be a target to reduce fatigue in the hemato‐oncological population (Allen et al., 2008). For example, moderate‐to‐high intensity aerobic exercise training has been shown to improve mitochondrial function and aerobic capacity and might thereby have the potential to improve muscle relaxation times (Allen et al., 2008). Supportively, moderate intensity aerobic exercise alone or combined with resistance exercise has shown to be effective in reducing perceived fatigue in hemato‐oncological populations (Campbell et al., 2019). However, it is important to note that exercise is likely to be beneficial for all populations, regardless of the specific role of muscle contractile properties. Furthermore, whether the exercise‐induced reduction in fatigue is indeed mediated by muscular mechanisms remains to be elucidated.

A strength of this study is the use of a unique and relatively large dataset containing identical and detailed electrical stimulation measurements in different populations allowing for direct comparison. A limitation of this study is the cross‐sectional study design which hampers the ability to draw conclusions on causal mechanisms between muscle contractile properties and perceived fatigue. In addition, the current study setup does not allow for insights into central mechanisms (α‐motorneuron activity) or more fundamental muscular mechanisms (muscle fiber type composition, mitochondrial function, or Ca^2+^ handling), which could contribute to fatigue. Additionally, the recordings of perceived fatigue were standardized within studies, but not across studies, potentially introducing variability. However, both fatigue questionnaires have a recall period of at least 24 h, which might limit within‐day variability. Nevertheless, as with any questionnaire, they may have been prone to recall bias. Similarly, not all VO_2_max measurements are conducted using gold‐standard techniques. The variability introduced by combining maximal and submaximal exercise tests might hamper comparability between groups and detectability of associations. Finally, the small sample size or little variation in perceived fatigue in some subpopulations may have hampered the detection of associations between contractile properties and perceived fatigue in these populations.

### 4.1 Perspective

By presenting detailed information about muscle contractile properties and their association with fatigue in various populations, this study has important clinical implications. Different mechanisms underlying perceived fatigue might be present in these populations, emphasizing the need for targeted treatment approaches. Future research might focus on causal mechanisms and the optimization of targeted exercise prescriptions in terms exercise type and dosing, to improve exercise capacity and reduce fatigue, across different study populations.

## 5 CONCLUSION

The present study demonstrates differences in physical fitness and muscle contractile properties of distinct patient populations and controls. Compared with a control group, patients with COPD and cancer survivors had a lower physical fitness. Also, we demonstrated that muscle contractile properties were not significantly associated with perceived fatigue in the total population of participants, but we found profound differences in associations between population subgroups. Specifically, in hemato‐oncological populations we found that impaired muscle relaxation was associated with higher perceived fatigue.

## AUTHOR CONTRIBUTIONS

IM, NA, DtH, BK, MH, and LB were involved in the conception of the study design. IM, NA, LJ, AS, BK, and DtH were involved in data collection. IM and LB analyzed the data and were responsible for initial writing and drafting the manuscript. IM, NA, DtH, JvH, AS, LJ, ST, BK, MH and LB discussed the results and contributed to the final manuscript. The final manuscript was approved by all authors.

## FUNDING INFORMATION

The contribution of LB and IM is supported by the Hypatia Fellowship grant from Radboud University Medical Center .

## CONFLICT OF INTEREST STATEMENT

Authors declare no conflicts of interest. The results of the study have been presented clearly, honestly, and without fabrication, falsification, or inappropriate data manipulation.

## ETHICS STATEMENT

All studies have been approved by the local medical ethics committee (METC Oost‐Nederland, formerly known as CMO region Arnhem‐Nijmegen)[13‐18], and all patients provided written informed consent. All procedures were conducted in accordance with the declaration of Helsinki.

## Supporting information


Table S1.

## Data Availability

The datasets generated during and/or analyzed during the current study are available from the corresponding author on reasonable request.
