# Supplementary material for: Muscle contractile properties and perceived fatigue in the general and diseased population
Source: Physiol Rep. 2024 Dec 11;12(23):e70134. doi: 10.14814/phy2.70134 (PMC11634544; doi:10.14814/phy2.70134)
Supplement: Supplementary file 1 — Table S1. [file PHY2-12-e70134-s001.docx]

| **Supplemental digital content 1. Differences in physical fitness and muscle contractile properties between patient populations and the control group** | | | | |
| --- | --- | --- | --- | --- |
|  | **Cancer Survivors**  **(n = 27)** | **Patients with CML**  **(n = 20)** | **Patients with COPD**  **(n = 16)** | **Statin users**  **(n = 64)** |
|  | β (95%CI) | β (95%CI) | β (95%CI) | β (95%CI) |
| **Body Mass Index** (kg/m^2^) | 1.2 (-0.9;3.3) | 0.1 (-2.2;2.5) | -0.3 (-2.7;2.1) | 1.0 (-0.6;2.6) |
| **Perceived fatigue** (0-100) | **37.9 (28.8;46.9)** | 4.3 (-6.0;14.7) | **34.7 (24.2;45.2)** | 5.7 (-1.1;12.6) |
| **PeakVO_2_** (ml/kg/min) | **-9.9 (-15.7;-4.1)** | -3.6 (-9.8;2.6) | **-20.9 (-27.2;-14.6)** | **-5.5 (-11.0;-0.1)** |
| **Maximal MVC** (N) | **-129.8 (-211.9;-47.8)** | 16.7 (-75.4;108.8) | **-148.3 (-243.3;-53.4)** | 23.1 (-39.4;85.6) |
| **MVC per kg bodyweight** (N/kg) | **-1.8 (-2.7;-0.9)** | 0.0 (-1.0;1.1) | **-1.7 (-2.8;-0.7)** | -0.1 (-0.8;0.6) |
| **Muscle force decline** (%) | -2.7 (-8.4;3.1) | -6.4 (-12.8;0.0) | **-10.5 (-16.7;-4.2)** | -3.0 (-7.2;1.2) |
| **Half relaxation time** (ms) | 3.4 (-1.7;8.5) | 3.8 (-1.7;9.3) | 3.1 (-2.3;8.4) | 0.6 (-3.1;4.3) |
| **Increase in half Rt** (%) | -13.1 (-49.2;22.9) | -8.6 (-47.4;30.3) | **83.1 (45.5;120.7)** | 14.4 (-11.7;40.5) |
| **Early Rt** (ms) | -2.8 (-9.1;3.6) | -3.5 (-10.7;3.6) | -3.1 (-9.9;3.8) | **-5.4 (-10.0;-0.8)** |
| **Increase early Rt** (%) | 16.5 (-13.7;46.7) | 10.9 (-23.0;44.9) | **84.4 (51.7;117.0)** | **26.2 (4.3;48.2)** |
| **MFR** (%/ms) | 0.0 (-0.1;0.1) | 0.0 (-0.1;0.1) | **0.2 (0.1;0.3)** | 0.0 (0.0;0.1) |
| **Decrease in MFR** (%) | 7.1 (-3.2;17.4) | -0.4 (-12.0;11.1) | **-24.3 (-35.7;-13.0)** | 1.3 (-6.2;8.7) |
| Differences with the control group are tested using linear regression models corrected for age, sex and study allocation.  Abbreviations: CML; chronic myeloid leukemia, COPD; chronic obstructive pulmonary disease, MFR; Maximal Force Rise, MVC; maximal voluntary contraction, Rt; relaxation time, PeakVO_2_; maximal oxygen uptake. | | | | |
